# Initial alpha-1 antitrypsin screening in Turkish patients with chronic obstructive pulmonary disease

**DOI:** 10.55730/1300-0144.5665

**Published:** 2023-06-04

**Authors:** Seda Tural ÖNÜR

**Affiliations:** Department of Pulmonology, Yedikule Chest Diseases and Thoracic Surgery Education and Research Hospital, University of Health Sciences, Istanbul, Turkiye

**Keywords:** Alpha-1 antitrypsin deficiency, COPD, emphysema

## Abstract

**Background/aim:**

Alpha-1 antitrypsin (AAT) deficiency is associated with several types of pathology, and the reported effects of mutations in the ATT-encoding gene vary worldwide. No Turkish study has yet appeared. We thus explored the AAT status of Turkish patients with chronic obstructive pulmonary disease (COPD).

**Materials and methods:**

This prospective cross-sectional study included outpatients and inpatients treated from June 2021 to June 2022. Serum AAT levels were checked, and dry blood samples were subjected to genetic analysis.

**Results:**

Genetic mutations were found in 21 (3.52%) of 596 patients with prior and new COPD diagnoses treated in our pneumonology outpatient department. The mean serum AAT level was 114.80 mg/dL (minimum 19, maximum 209; standard deviation 27.86 mg/dL). The most frequent mutation was M/Plowell (23.8%, n = 5), followed by M/S (23.8%, n = 5), M/I (19%, n = 4), M/Malton (14.3%, n = 3), Z/Z (9.5%, n = 2), M/Z (4.8%, n = 1), and Kayseri/Kayseri (4.8%, n = 1). Thoracic computed tomography revealed that 85.7% (n = 18) of all patients had emphysema, 28.5% (n = 6) had bronchiectasis, and 28.5% (n = 6) had mass lesions. Of the emphysema patients, 55% (n = 10) had only upper lobe emphysema, and 83.3% (n = 15) had emphysema in additional areas, but statistical significance was lacking (p > 0.05).

**Conclusion:**

In patients with emphysema and normal serum AAT levels, genetic analyses may reveal relevant heterozygous mutations, which are commonly ignored. Most clinicians focus on lower lobe emphysema. Evaluations of such patients might reveal AAT mutations that are presently overlooked because they are not considered to influence COPD status.

## 1. Introduction

Alpha-1 antitrypsin (AAT) is secreted by cells of the liver, gastrointestinal and respiratory systems, neutrophils, and macrophages. Alpha-1 antitrypsin deficiency (AATD) is an autosomal-dominant condition that is severe when two alleles encoding the Serpina 1 protein are replaced to an extent of at least 95% by the Z allele [1,2]. Poor neutrophil elastase function causes the lung damage associated with AATD [3] and the inability to inhibit AAT-mediated lung oxidation and polymerisation. AAT also serves as a proinflammatory haemoattractant, as the resulting airway damage ultimately triggers emphysema [4]. The synthesis, secretion, and function of AAT may be pathologically compromised. The rate of Z allele homozygosity (ZZ) is 1/2000 in Europe but differs elsewhere. ZZ status reduces the AAT level, as do the Siiyama, M/Malton, and King alleles and possibly other heterozygous mutations. Milder cases (with the S and Null alleles) bear amino acid substitutions in different proteins, but the allelic frequencies are generally unknown and vary regionally [1]. Most relevant literature focuses on ZZ. Several cross-sectional cohort studies have shown that emphysema and liver fibrosis are associated with AATD [5–7]. Various mutations have been described in case reports. The level of the common MZ mutation, which is in the grey zone, is >4% in Europe [8]; neither the clinical course of patients with this mutation nor the regional incidence in the Mediterranean region are known. Because the disease is rare, Turkish data on AATD; even less is known about subjects heterozygous for various mutations. We thus screened patients with chronic obstructive pulmonary disease (COPD) for AATD and relevant mutations. Although a relationship between the development of COPD and MZ heterozygosity has not been clearly demonstrated, liver disease in such patients is associated with various risk factors, as is COPD itself [9–11].

## 2. Materials and methods

This prospective cross-sectional study was conducted in our pneumonology in- and outpatient departments from June 2021 to June 2022. All COPD patients were diagnosed with pulmonary function tests performed by a pneumonologist. Serum AAT levels were measured, and dry blood samples (DBSs) were subjected to genetic analysis. COPD cases with suspected AAT deficiencies were identified. The inclusion criteria were age over 40 years; COPD (diagnosed by a chest physician) with dyspnoea, chronic cough, or sputum production; a history of exposure to risk factors for COPD; a forced spirometry FEV1/FVC value <0.7; and a willingness to participate in the study. All patients had stable COPD and had not been diagnosed with AATD.

The exclusion criteria were the absence of a COPD diagnosis, an unwillingness to participate, and COPD accompanied by an infection or exacerbation that might increase the AAT level. Serum AAT is an acute phase reactant, increasing in patients with acute inflammation, cancer, and liver disease; we thus performed dry blood analyses. The AAT variants recognised by the American Thoracic Society/European Respiratory Society were divided into four clinical groups: “normal” indicates normal responses and a normal serum AAT level, “deficient” refers to a serum AAT level below 20 and concomitantly decreased functional activity, “null” indicates the absence of serum AAT, and “dysfunctional” indicates abnormal function [12]. If first-degree relatives (mother, father, siblings, and children) of a patient with a genetic mutation wished to be screened, we agreed.

The study adhered to the principles of the Declaration of Helsinki, and the protocol was approved by the Ethics Committee of the Health Sciences University İstanbul Yedikule Chest Diseases Training and Research Hospital (approval no. 2022-241).

GPower analyses were used to determine the required sample size, which was 111 for an effect size of 0.3, an error rate of 0.05, and a power of 95%.

### 2.1. Electronic data and laboratory analyses

Demographic data, medical histories, laboratory findings, and thoracic computed tomography (CT) data were obtained from our electronic data system. Laboratory data were obtained using blood taken on the first visit. Haemograms were obtained on a Beckman Coulter AU2700 platform, and biochemical data were obtained using a Sysmex XT4000i platform. All data were double-checked. Noninfectious/noncontagious DBSs were used for routine screening and initial diagnosis. We used serum samples and DBSs to screen and test for AATD using AlphaKits from GE Healthcare (Cardiff, UK). Genotyping was performed at the Progenika Biopharma laboratory in Spain. The AAT genotyping test used Luminex xMAP technology. Genomic DNA was extracted from DBSs, EDTA-anticoagulated whole blood, or buccal swabs, then amplified and biotinylated via multiplex PCR. The PCR products were denatured and hybridised to oligonucleotide probes coupled to colour-coded beads. Hybridised DNAs were labelled with fluorescent conjugates, and the resulting signals were detected with a Luminex 200 system. The raw data were processed with AAT Genotyping Test Analysis software, which converts variant allelic genotypes to the alleles of the current literature. Whole-blood samples that become DBSs are stable for up to 6 months when maintained at ambient temperature away from direct sunlight; contact between different specimens was avoided.

The lowest DNA concentrations at which 95% of sample replicates yielded correct results were determined by testing 20 replicates of several DNA dilutions of five genomic samples using two reagents “Lots.” The lowest limit of detection was 0.0310 ng/μL. The AAT genotyping results were compared to those of bidirectional Sanger sequencing. A total of 116 DNA samples, including as many variants as possible, were distributed as follows: 66 clinical samples, 46 genomic DNAs extracted from cell lines, and 4 synthetic DNA samples. This panel covered all heterozygous and homozygous genotypes of all allelic variants and 15 compound heterozygous genotypes. The AAT genotyping test and bidirectional Sanger sequencing data were in complete agreement.

### 2.2. Parameters

We recorded patient demographics, comorbid diseases, dates of outpatient admission with COPD diagnoses, mMRC dyspnoea scores, and any COPD exacerbations. The following values were recorded at admission: a complete blood count (CBC), the international normalised ratio (INR), serum aspartate aminotransferase and alanine aminotransferase levels, renal function test data, the serum AAT level, pulmonary function test data, and CT findings.

### 2.3. Statistical analysis

SPSS version 21.0 was used for all statistical analyses. Descriptive statistics are presented as means with standard deviations, frequencies, or percentages and were compared with the chi-square test. We evaluated the normality of the distribution using the Shapiro–Wilk test. The Mann–Whitney U test was used to compare the two groups because the data were not normally distributed. p < 0.05 was taken to reflect statistical significance.

## 3. Results

**Figure 1:**
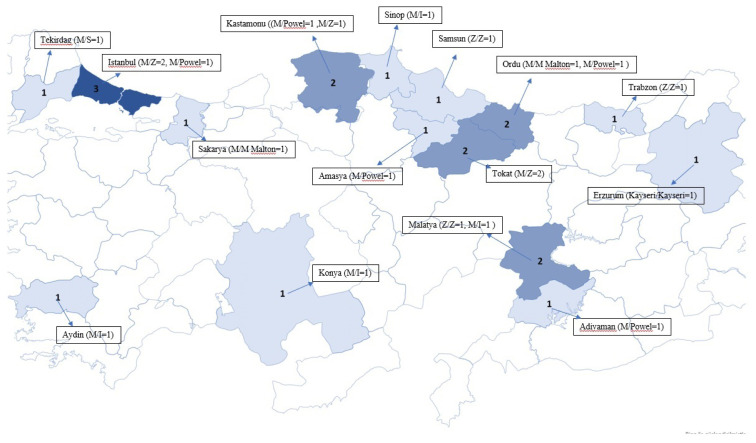
Distribution of mutation analysis results on Turkey map.

Relevant mutations were detected in 21 (3.52%) of 596 patients with previous and new-onset COPD admitted to the pneumonology outpatient clinic. Genetic mutations were found in 17 (40.47%) of 42 relatives who agreed to participate in the study. The most common mutations in relatives were M/S (n = 13), M/Plowell (n = 3), and Z/Z (n = 1). The highest positivity rate was that of relatives of Z/Z patients (63.7%–100%); the positivity rate was 20% in the M/Malton group and 21.4%–28.5% in the M/Z group. Figure 1 shows the mutations in patients. The M/Z mutation was most common in the relatives of patients with Z/Z mutations (88.8%, eight of nine M/Z subjects). The mutational analyses of patients and patients plus relatives are listed in Table 1. The most common mutation in patients (n = 5) was M/Z (n = 1). The most common mutations in relatives were M/Z (n = 18) and M/Plowell AAT (n = 8). The distribution of mutations in Türkiye is shown in the map in Figure 1.

Of the patients, 19 were male (90.5%), and the mean age was 59.19 years (range 34–82 years; SD 14.4 years). Three (14.3%) were active smokers, 16 (76.2%) were ex-smokers, and 2 (9.5%) had no history of smoking. Of the relatives, 28 (73.7%) were male, and the mean age was 49 years (range 6–82 years). The mean serum AAT level of patients was 114.80 mg/dL (range 19–209 mg/dL; SD 27.86 mg/dL). The mean value of for all patients was 104 mg/dL. Although no mutation significantly affected the serum AAT level (p = 0.054), 80% of patients with M/Plowell, 100% of those with M/I, and 100% of those with M/S had levels below the normal limit (Table 2).

In terms of the COPD phenotype, 1 patient was a chronic bronchitic, 2 exhibited frequent exacerbations, and 18 had the emphysematous phenotype. The AAT level was low in 67% of the latter group. There were no significant differences among the phenotypes because of the small numbers of cases in the other groups.

**Figure 2:**
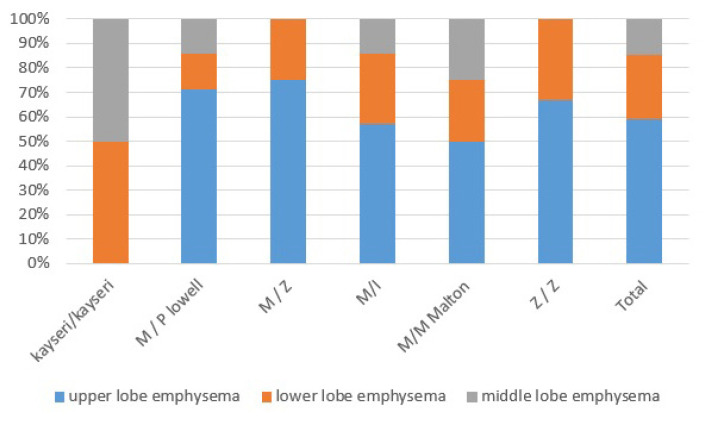
The distribution of emphysema in computed tomography of the thorax by mutation types.

The mean FEV1 of 17 patients was 1.4 L (0.4–2.7 L); we found no correlation between the AAT level and FEV1 (p > 0.05). On thoracic CT, 85.7% of patients (n = 18) had emphysema, 28.5% (n = 6) had bronchiectasis, and 28.5% (n = 6) had mass lesions. In terms of emphysema, 55% (n = 10) had only upper lobe emphysema and 83.3% (n = 15) had emphysema in other areas (Figure 2). There was no significant association between emphysema and smoking status (p > 0.05). Although no significant association was found between the AAT level and mutational status, the AAT level was within normal limits in 80% (n = 4) of the M/Plowell group and 100% (n = 4) of the M/I group. All patients (n = 4 and n = 4) in the M/Plowell and M/I Malton groups with AAT levels within normal limits had COPD, were receiving bronchodilator therapy, and exhibited extensive emphysema on thoracic CT. When the C-reactive protein (CRP) and AAT levels were compared, the latter was below the normal limit (>90 mg/dL) in 4 of 15 patients (26.6%) with CRP values above the normal limit (>5 mg/dL), whereas 4 of 6 patients (66.6%) had normal CRP levels but AAT levels below the normal lower limit.

## 4. Discussion

It is difficult to distinguish nonhereditary emphysema and emphysema caused by AATD. Most AATD-related cases are diagnosed only when symptoms become more obvious in later decades of life [1]. In addition, patients with heterozygous mutations are usually diagnosed simply with “COPD” in outpatient clinics; the number of misdiagnosed cases cannot be estimated. Here we found that 21 (3.52%) of 596 COPD patients admitted to our outpatient clinic, which is a national reference centre, had AATD; this is a higher incidence than that of our closest geographic neighbour. Apart from the Z/Z, M/M Malton, and M/Plowell mutations, which are very damaging, M/I and M/S mutations were common. AATD were first described in 1963 in three of five young patients with emphysema [13]. Although early lower lobe emphysema reflects cigarette use in severe cases, the radiological presentation is upper lobe bronchiectasis in more than 37% of patients with severe AATD [14], AATD status should be investigated in all COPD patients [1,12,15]. The radiological findings of stable patients visiting our outpatient clinic were in line with those in the literature. We emphasise that emphysema associated with heterozygous mutations is not lower lobe dominant.

AATD is an acute phase reactant and should be measured at the same time as CRP, but it is normally high in heterozygous patients. We found that most heterozygous patients had normal AAT levels but suffered from COPD. We emphasise that if a patient is heterozygous and the AAT level is normal, close follow-up is essential; when the AAT level decreases, replacement therapy should commence immediately [16]. Nonpathogenic MZ and MS heterozygous mutations increase the risk for lung disease in smokers [1,9,17], those exposed to environmental pollutants, and those with a genetic predisposition. We are deriving an accurate treatment algorithm for irreversible OPD in young patients [12]. Early diagnosis protects such patients from COPD and COPD-related conditions caused by life-changing environmental risk factors. In such cases, we reduce exposure to such factors and prescribe replacement therapies [18]. We emphasise that international large-scale studies are required.

Scavenger et al. conducted the largest population-based study to date of autosomal codominant inherited AAT deficiency identified via a severe deficiency of AAT in Swiss newborns; the prevalence was 1/1639 [19]. When the results of O’Brien et al. were extrapolated to the entire American population, it was estimated that approximately 63,000 had severe AATD [20,21]. Serres, in 2015, extrapolated 2002 data to the entire world population and estimated that 5.64 million persons had AATD [21]. The 2022 figure is unknown; we believe that we are only seeing the tip of the iceberg. The Z allele is more common in northern Europe than elsewhere and is rare in Asians [19,22]. Data on Africans are lacking, but Z is less common among Africans and Hispanics than Whites living in America [23]. Given DNA transmission along the Silk Road, which ran through Turkey, there is much work to do. We found that the number of undiagnosed patients is very high; the rate of heterozygous mutations is 8%. Physicians are not familiar with AATD, patients are diagnosed late because symptoms appear late, and very few patients are screened [2]. Early diagnosis, reduced environmental exposure, and individualised treatments for COPD and other disorders will reduce the burdens on health systems [19,24].

In the retrospective analysis of Bornhorst et al., frequency was 80.4% for the MM allele, 7.6% for MS, 7.1% for MZ, 1.1% for ZZ, and 0.74% for SZ [25]. A recent report on diagnosis that included 1936 COPD cases showed that genetic mutations originating in Turkey are rare, thus 0.4% (7) MS, 1.8% (34) MZ, and 0.3 (6) ZZ cases, but the family screening rate was low [26]. Our figures were M/Plowell 23.8% (n = 5), M/S 23.8% (n = 5), M/I 19% (n = 4), M/Malton 14.3% (n = 3), Z/Z 9.5% (n = 2), M/Z 4.8% (n = 1), and Kayseri/Kayseri 4.8% (n = 1). In our study, the most common symptom was dyspnoea, consistent with a National Heart, Lung, and Blood Institute Report; the mean patient age was 59.19 years; and most patients had been diagnosed late [27]. The average interval between the onset of pulmonary symptoms and diagnosis was 8.3 years.

## 5. Limitations

This study was conducted at a regional reference hospital, and thus our results cannot be generalised to the entire Turkish population with COPD. A multicentre study is required. AATD is an acute phase reactant after inflammation, and measurement is laboratory dependent. However, we also tested DBSs. In addition, we did not perform multivariate analyses.

As serum AAT is an acute phase reactant, we performed simultaneous dry blood analyses because the AAT level increases during acute inflammation, cancer, and liver disease. Diagnosis was simple in patients with exacerbations. Patients with advanced respiratory failure require more than routine outpatient control and are hospitalised. We believe that screening DBSs during hospitalisation would be useful. We believe that if a patient with emphysema has a normal serum AAT level, genetic analyses should follow, because the patient may have a heterozygous mutation. Multicentre data collection might detect unknown AAT mutations presently overlooked because of COPD diagnoses.

## Figures and Tables

**Table 1:** Mutation analysis of patients and their relatives.

	Patient		Patient and relatives of the patient	
Mutations	Frequency (n)	Percent (%)	Frequency (n)	Percent (%)
Kayseri/Kayseri	1	4.8	1	2.6
M/P lowell	5	23.8	8	21.6
M/Z	1	4.8	1	2.6
M/S	5	23.8	18	47.6
M/I	4	19	4	10.5
M/M Malton	3	14.3	3	7.9
Z/Z	2	9.5	3	7.9
**Total**	21	100	38	100

**Table 2:** Serum AAT levels according to mutation results.

Serum AAT level	Mutation analysis							Total
	Kayseri/Kayseri	M/P lowell	M/S	M/Z	M/I	M/M Malton	Z/Z	
**Normal** *	**0**	4	1	2	4	2	0	13
**Low** ^**^	**1**	1	0	3	0	1	2	8
**Total**	1	5	1	5	4	3	2	21

* Serum AAT level Normal is >90 mg/dL, Serum AAT level low is <90 mg/dL
